# Thrombocytopenia: incidence and clinical impact on ICU mortality

**DOI:** 10.1186/cc9861

**Published:** 2011-03-11

**Authors:** M Kompoti, M Michalia, P Peppa, F Nanou, M Charitidi, M Sartzi, I Vassiliadis, G Kallitsi, P Clouva-Molyvdas

**Affiliations:** 1 Thriassio General Hospital of Eleusis, Athens, Greece

## Introduction

The prognostic significance of thrombocytopenia in critically ill patients has not been thoroughly explored. Our study aimed at investigating the incidence of thrombocytopenia among ICU patients, its association with ICU-acquired infection and its clinical impact on ICU mortality.

## Methods

All patients admitted to the ICU were prospectively followed with daily platelet (PLT) count measurement until ICU outcome. Thrombocytopenia was defined as PLT count lower than 150,000/mm^3 ^and severe thrombocytopenia as PLT count lower than 20,000/mm^3^. Data were analyzed with one-way ANOVA and logistic regression with statistical significance set at *P *< 0.05.

## Results

We studied 169 consecutive patients (119 males, 50 females) aged (mean ± SD) 53.4 ± 19.8 years, with admission APACHE II score 22.7 ± 5.3. Thrombocytopenia during ICU stay was recorded in 101 patients (59.8%). Emergency surgical and trauma patients displayed the highest incidence of thrombocytopenia (77.3% and 72.1%, respectively). Emergency surgical and medical patients displayed the highest of severe thrombocytopenia (18.2% and 10.6%, respectively). Trauma and emergency surgical patients developed thrombocytopenia earlier during the ICU stay (that is, after 4.9 and 5.1 days, respectively) compared with medical and elective surgical patients (that is, after 13.3 and 10.9 days, respectively) (*P *= 0.001). Thrombocytopenia was more often recorded in patients with ICU-acquired infection compared with patients without infection. In particular, severe thrombocytopenia was recorded in 18.9% of patients with bloodstream infection and 9.0% of patients with other ICU-acquired infection. ICU mortality was significantly higher in patients who developed thrombocytopenia compared with patients with normal PLT counts throughout the ICU stay (30% vs. 9.4%, *P *= 0.002). In a logistic model adjusted for age, gender, admission diagnosis, admission APACHE II score and the occurrence of ICU-acquired infection, thrombocytopenia was independently associated with ICU mortality (*P *= 0.017) and the degree of PLT count decrease significantly increased the ICU mortality in a dose-dependent manner; that is, odds ratios of 3.4 for PLT 100,000 to 150,000/mm^3^, 3.5 for PLT 50,000 to 100,000/mm^3^, 14.9 for PLT 20,000 to 50,000/mm^3 ^and 25.2 for PLT below 20,000/mm^3^.

## Conclusions

Thrombocytopenia was a common finding in our sample of ICU patients. Although the time of occurrence and the degree of PLT count decrease varied, reflecting a wide spectrum of pathogenic mechanisms, thrombocytopenia was independently associated with ICU mortality in a dose-dependent manner.

